# *Staphylococcus pseudintermedius* septicemia in puppies after elective cesarean section: confirmed transmission via dam’s milk

**DOI:** 10.1186/s12917-019-1795-y

**Published:** 2019-01-28

**Authors:** Maja Zakošek Pipan, Tanja Švara, Irena Zdovc, Bojan Papić, Jana Avberšek, Darja Kušar, Janko Mrkun

**Affiliations:** 1 Clinic for Reproduction and Large Animals, Veterinary Faculty, University of Ljubljana, Gerbičeva 60, 1000 Ljubljana, Slovenia; 2 Institute of Pathology, Wild Animals, Fish and Bees Veterinary Faculty, University of Ljubljana, Gerbičeva 60, 1000 Ljubljana, Slovenia; 3 Institute of Microbiology and Parasitology, Veterinary Faculty, University of Ljubljana, Gerbičeva 60, 1000 Ljubljana, Slovenia

**Keywords:** *Staphylococcus pseudintermedius*, Puppies, Canine milk, Whole genome sequencing

## Abstract

**Background:**

In humans, transmission of bacteria causing fatal sepsis in the neonates through mother’s milk has been reported. In dogs, it is believed that bacteria from canine milk are not the primary cause of neonatal infections. *Staphylococcus pseudintermedius* is colonizing the skin and mucocutaneous junctions in adult dogs and can act as an opportunistic pathogen. This bacterium was previously isolated from the canine milk and, although, its transmission from the dam’s milk to the newborn puppies causing a neonatal sepsis was suggested, this hypothesis has not been confirmed.

**Case presentation:**

A 4.5-year-old healthy Boston terrier dam had an elective cesarean section, delivering five normal puppies and one dead runt. Next day, two puppies developed pustules on their legs and around the muzzle. After two more days, strings of blood were noticed in the stool of the biggest puppy that suddenly died later that night. The same day, blood became visible in the feces of all other puppies. Necropsy of the dead puppy revealed a distended abdomen, catarrhal gastroenteritis with lymphadenopathy, dark red and slightly firm lung, mild dilatation of the right heart chamber and congestion of the liver, spleen, pancreas and meninges. The thoracic cavity contained white-yellow slightly opaque exudate, and there was transudate in the abdominal cavity. Histopathology revealed an acute interstitial pneumonia and multifocal myocardial necrosis with mineralization. Bacteriology of the internal organs, body cavity effusions of the dead puppy and dam’s milk revealed a diffuse growth of *S. pseudintermedius* in pure culture. Whole genome sequencing (WGS) revealed that all isolates belonged to the sequence type 241 and differed in 2–5 single nucleotide polymorphisms; thus, the epidemiological link between the outbreak-associated isolates was confirmed.

**Conclusions:**

This is the first report of a confirmed transmission of *S. pseudintermedius* through dam’s milk causing a neonatal sepsis in a puppy after an elective cesarean section. The epidemiological link between *S. pseudintermedius* isolates obtained from dam’s milk and internal organs of the affected puppy was confirmed by WGS. Our findings indicate that milk of healthy dams can serve as a reservoir of bacteria that can cause fatal sepsis in the newborn puppies.

## Background

The mortality rate in puppies from birth until 7–8 weeks of age is reported to account for 20% of all newborns [1, 2]. Most deaths (75–90%) occur during the first 2–3 weeks of life [3–5] and sepsis is the major cause of mortality in these puppies. At birth, due to the immunocompromised status, puppies are susceptible to infection with bacteria that are generally regarded as commensal colonizers of the mucosal surfaces and skin. The most common bacterial species implicated in these infections are members of the family *Enterobacteriaceae* (e.g. *Escherichia coli*, *Klebsiella* spp., *Proteus* spp., *Salmonella* spp. and β-streptococci) [6]. In dogs, *Staphylococcus pseudintermedius* is often identified as a commensal colonizer, but has been also associated with opportunistic infections such as pyoderma in the immunocompromised dogs [7]. This bacterium has been rarely identified as the cause of a neonatal mortality in puppies. In the three reported cases where puppies died in the first two weeks of life due to *S. pseudintermedius* infection, septicemia, omphalophlebitis with multifocal abscessation and dermatitis/pododermatitis were diagnosed [8–10]. In most of these cases, the clinical signs started with skin lesions leading to a systematic spread of *S. pseudintermedius*. Transmission of *S. pseudintermedius* from the dam’s milk to the newborn puppies was mentioned as a potential cause of the neonatal sepsis but it was not confirmed. It was concluded that abrasion of the skin by rough surfaces during the feeding activity permits *S. pseudintermedius* to proliferate and cause lesions on the foot pads and omentum, and that staphylococcal dermatitis (‘black spot disease’) should be recognized as the cause of canine neonatal mortality [10]. Other authors suggested that bacteria from the milk of healthy dams are usually not the primary cause of neonatal infections [11].

The case report presented herein adds to the evidence that *S. pseudintermedius* can be the causative agent of a fatal neonatal sepsis in puppies born with cesarean section (C-section) via transmission through the dam’s milk to the newborns. To the best of our knowledge, this is the first report in which the transmission of *S. pseudintermedius* from the canine mother to the puppies through breast milk was confirmed by whole genome sequencing (WGS).

### Case report

**Fig. 1:**
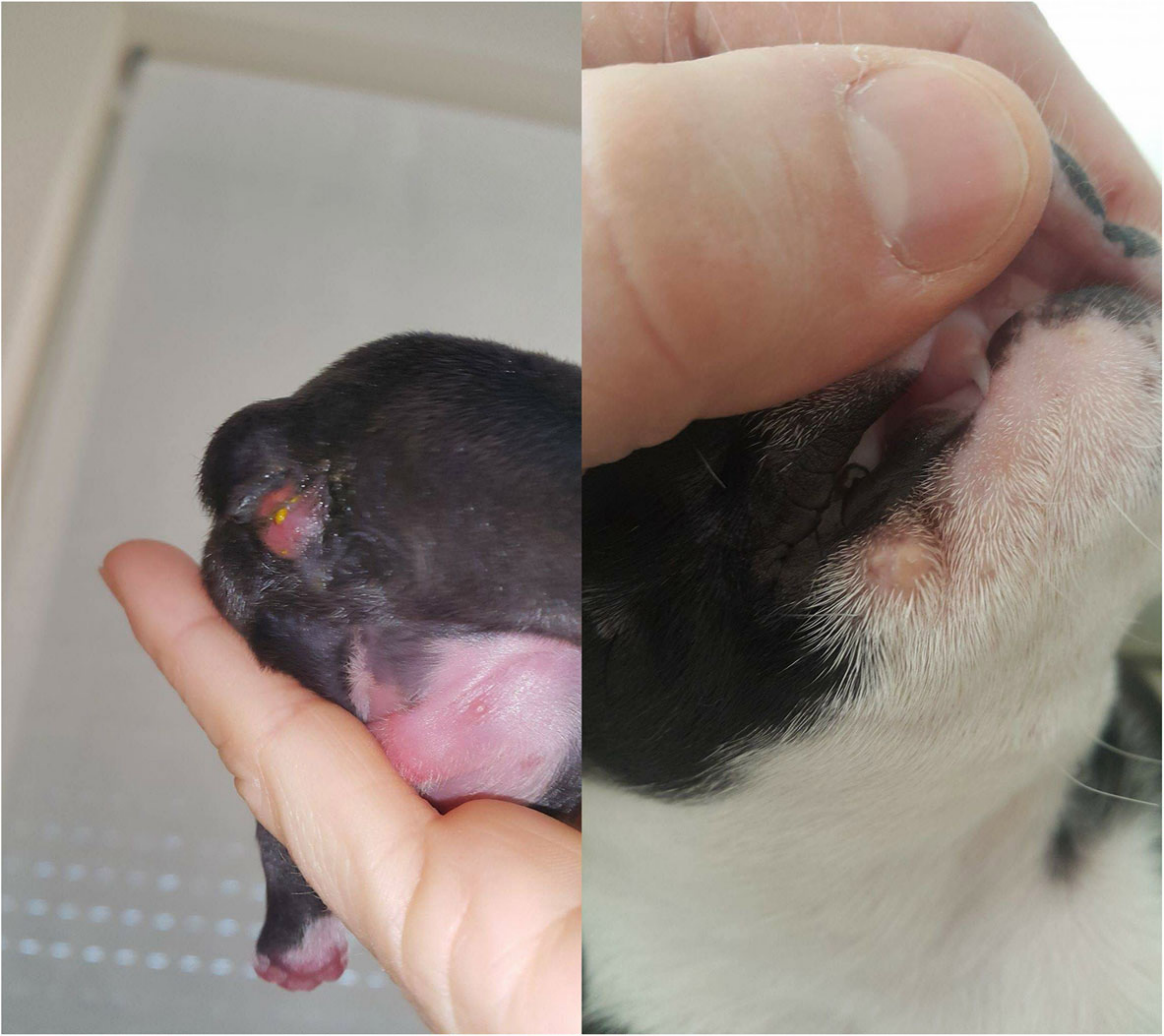
Left: Yellowish diarrhea with a few strings of blood visible in the feces of the puppy. Slightly blue discoloration is visible on the abdominal skin and foot of the puppy. Right: Pustules around the muzzle in a one-day old puppy

A 4.5-year-old Boston terrier dam, properly vaccinated (against canine distemper virus, canine parvovirus, canine adenovirus, canine parainfluenza virus, leptospirae and herpesvirus) and regularly dewormed, had an elective C-section. It was systemically healthy with a normal coat and no visible skin lesions, and showed no signs of mastitis. In the last year, it was not treated with antibiotics, but was bathed with Betadine shampoo at least once per month. Its complete blood count and chemistry did not reveal any abnormalities. Reports on the previous reproductive disorders were absent and the hygienic and sanitary conditions of the household were adequate; ultrasound of the reproductive organs did not show any abnormalities. Before the surgery, the dam was preoxygenated for 5 min before anesthesia that was induced by administration (4.4 mg/kg BW IV) of propofol (PorpoFlo, Zoetis, Slovenia) and followed by sevoflurane maintenance. The induction was followed by the application (0.2 mg/kg BW SC) of methadone after skin incision. The C-section was performed by a midline incision of the abdomen from the pubis to the umbilicus and a ventral incision on the uterine body to allow a quick removal of the fetuses. Five normal puppies and one dead runt puppy were delivered. Immediately after the birth, the modified Apgar score [12] reflected an initial depression of vital functions in all five puppies. An adequate recovery occurred within 15 min in four puppies that were included into the healthy group. The biggest puppy remained moderately stressed, but his Apgar score normalized in one hour. One day after the birth, two puppies developed pustules on their legs and around the muzzle (Fig. 1). Three days after the birth, a small amount of fresh blood was noticed in the stool of the biggest puppy, which then suddenly died during the night (Fig. 1). On the same night, a small amount of blood became visible in the feces of the remaining puppies. During the clinical examination, cyanosis, bradycardia, swelling and distension of the abdomen, slightly blue discoloration of the abdominal skin and normal chest sounds were noticed in all the remaining puppies.

A supportive therapy with amoxicillin and clavulanic acid (Clavamox, Zoetis, Slovenia; 20 mg/kg/12 h PO) for 10 days, intraosseous fluid therapy (6 ml/kg/h) and oxygen support were commenced. After one day of the therapy and feeding with formula, all puppies improved (normal pulse, heart sounds, breathing without oxygen support, no bloating, normal bowels and normal appetite) and were discharged home. However, after a few hours of suckling mother’s milk, puppies started to vocalize, became bloated and showed signs of a painful abdomen. The supportive treatment with antibiotics, IV fluids and oxygen was reinstituted. Additionally, puppies were separated from the dam and put back onto the formula for the whole week. Milk samples from the dam were collected for the bacteriological examination. The antimicrobial therapy with amoxicillin and clavulanic acid (Clavamox, Zoetis, Slovenia; 12.5 mg/kg/12 h PO) was implemented for the dam. Three days after dam’s antibiotic treatment, puppies were allowed to suckle its milk again and no further deterioration appeared. At the end of the antimicrobial treatment, there was no bacterial growth observed from the dam’s milk. At the time of writing, puppies are 1.5-year old and systemically healthy. No signs of recurrent *S. pseudintermedius* infection have been noted since.

The following year, for the same dam, the owners decided to have another litter of puppies. The vaginal and skin swabs were collected before the artificial insemination. A diffuse growth of *S. pseudintermedius* was diagnosed in both samples*.* Due to the absence of clinical signs, the antimicrobial treatment was not prescribed. Repeated samples for the bacteriological examination were collected a week before the elective C-section from the vagina, skin and milk. A diffuse growth of *S. pseudintermedius* was still present in all samples and amoxicillin with clavulanic acid (Clavamox, Zoetis, Slovenia; 12.5 mg/kg/12 h PO) was prescribed for one week. Puppies were born healthy with elective C-section and did not have any health issues; after weaning, their monitoring was discontinued.

### Specimen collection

For the bacteriological examination, milk samples, vaginal swabs and skin swabs were collected. Before collecting the milk samples or vaginal swabs, nipples or perivulvar area were cleaned with the chlorhexidine wipes (PetMD, USA), respectively. The first three streams of milk were discharged. Milk from four different mammary glands was collected into a sterile vial without touching the nipples or skin. For vaginal samples, a sterile double-guarded cotton swab (Minitube, Germany) was inserted through a vaginal speculum to avoid contamination and gently rotated for 15–30 s. The skin swabs were collected from four different sites: ventral area around the thoracic and abdominal mammary glands, right dorsal interdigital skin between digits IV and V, and dorsal perineal area. Each swab was rubbed on the skin 40 times, while rotating each swab by one quarter for every 10 strokes.

### Necropsy

During the necropsy, samples of brain, lung, spleen, heart, liver, kidney, stomach and intestine were collected for histopathological examination, fixed in 10% buffered formalin and embedded in paraffin. Tissue sections of 4 μm were deparaffinized and stained with hematoxylin and eosin (HE). Samples of the small intestine, liver, lung, spleen, and pleural and peritoneal effusions were collected for the bacteriological examination. For virological examination, samples of liver, lung, spleen and lymph nodes were collected, and the intestine was sampled for parasitological examination.

**Fig. 2:**
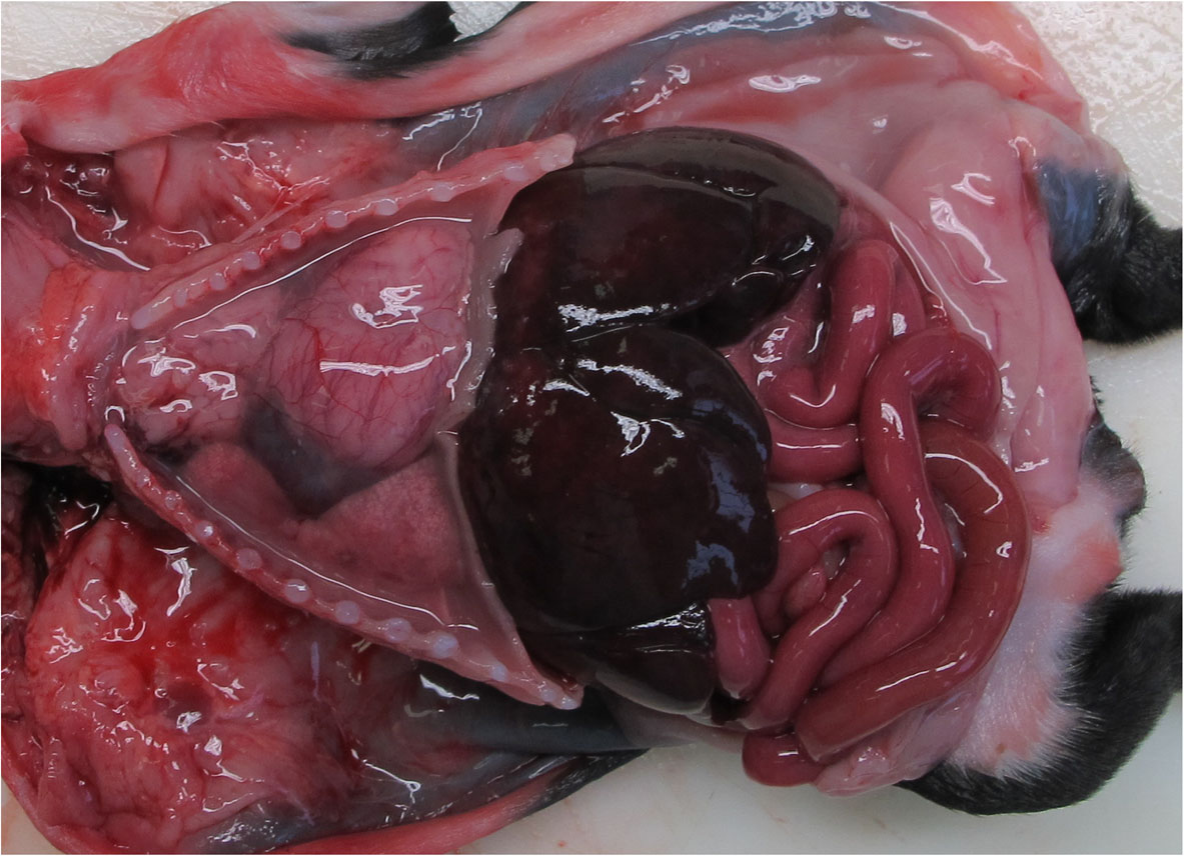
Thoracic and abdominal cavity of the deceased puppy. The necropsy revealed catarrhal gastroenteritis, congestion of the liver and white yellow, slightly opaque exudate in the thoracic cavity

**Fig. 3:**
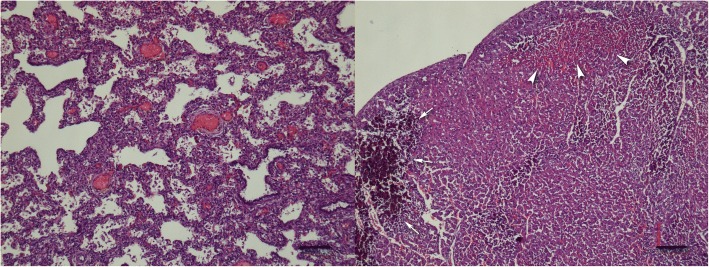
Left: Histology picture of lungs: Acute interstitial pneumonia. Scale bar, 100 μm. Right: Histology picture of myocardium: Myocardial necrosis (arrowheads) and mineralization (arrows) of the left ventricle. Scale bar, 100 μm

Necropsy of the deceased puppy revealed a catarrhal gastroenteritis with lymphadenopathy of mesenteric lymph nodes (Fig. 2). The small intestine was mildly filled with yellow dense contents, and the large intestine contained a small amount of green-yellow dense contents. The congestion of liver, spleen, pancreas and meninges was observed. Thoracic cavity contained white-yellow slightly opaque exudate. Lungs were dark-red and slightly firm, and mild dilatation of the right heart chamber was noticed. Histopathological examination confirmed gross findings. The mucosa of the stomach and small intestine was covered with desquamated epithelial cells and mucous, and coccoid and rod-shaped bacteria were observed in the small-intestine contents. There was a diffuse loss of apical enterocytes and multifocal loss of enterocytes in the intestinal crypts. The acute interstitial pneumonia, multifocal acute myocardial necrosis, and multifocal dystrophic myocardial mineralization were also observed (Fig. 3). Portal areas of the liver were edematous and infiltrated with histiocytes and lymphocytes. Even though the dam was vaccinated according to the protocol, samples were collected for the determination of parvovirus and canine distemper virus due to the myocardial necrosis and mineralization, but were PCR-negative. The parasitological examination was also negative.

### Bacteriology and antimicrobial susceptibility testing

All samples were inoculated onto nutrient agar plates (Oxoid, UK) supplemented with 5% of sheep blood and Sabouraud dextrose agar plates (Oxoid, UK) with chloramphenicol (100 mg/l); they were incubated at 37 °C for up to three days. The isolated colonies were identified to the species level using the MALDI-TOF system (Bruker, Germany). On Sabouraud dextrose agar plates, the pathogenic fungi were not detected.

The skin and milk samples were further examined for the methicillin-resistant staphylococci (MRS) by a selective enrichment according to the laboratory protocol of the EU Reference laboratory for antimicrobial resistance (DTU Food, Copenhagen, Denmark). The swabs were soaked in 5 ml of Mueller-Hinton Broth (Oxoid, UK) supplemented with 6.5% NaCl, and incubated at 37 °C for 16–20 h. One ml of the enriched broth was inoculated into 9 ml of tryptone soya broth (TSB; Biolife, Italy) supplemented with 35 mg/l cefoxitin and 75 mg/l aztreonam, and incubated for additional 16–20 h at 37 °C. One loop-full (10 μl) of TSB culture was spread onto the selective agar plate (Brilliance MRSA agar, Oxoid, UK) and blood agar plate; they were incubated for 24–48 h at 37 °C. Presumptive MRS colonies were subcultivated on blood agar plates.

Bacteriological examinations of all internal organs and effusions from the dead puppy and dam’s milk showed a diffuse growth of *S. pseudintermedius* in pure culture. The dam’s skin was also positive for *S. pseudintermedius* as few colonies were observed. In addition, *Clostridium perfringens* was isolated as few colonies were identified from the puppy’s liver, kidneys, spleen and abdominal cavity effusion.

In addition, all *S. pseudintermedius* isolates were tested for their antibiotic susceptibility and the minimal inhibitory concentrations (MICs) were determined. The testing was performed using the commercially available 96-well broth microdilution plates for staphylococci (EUST format, Trek Diagnostic Systems, UK). The 19 antimicrobial agents tested are listed in Table 1. Testing was performed as recommended by the manufacturer, using the cation-adjusted Mueller-Hinton broth with TES (Trek Diagnostic Systems, UK) to prepare the inoculum containing 1 × 10^5^ to 1 × 10^6^ CFU/ml. The plates were incubated at 35 °C for 24 h in aerobic conditions. The MIC endpoints were determined where no growth or the most significant reduction of growth was observed. Although oxacillin is the prescribed indicator antibiotic for the methicillin-resistant *S. pseudintermedius* (MRSP), it is not included in the commercial plate; therefore, it was tested separately by the classical disc diffusion method [13] using the Mueller-Hinton agar plates (Merck, Germany). *Staphylococcus aureus* ATCC 29213 reference strain was used for the quality control of MIC procedure. Results of the MIC determination for the obtained *S. pseudintermedius* isolates are given in Table 1.

**Table 1:** Antimicrobial sensitivity testing using a microdilution method to determine the minimal inhibitory concentration (MIC), except in additional oxacillin^*^ testing, in which the disk diffusion method was used; all the tested *Staphylococcus pseudintermedius* isolates showed the same pattern of resistance

Antimicrobial	Results and interpretation		
	MIC value (μg/ml)		S/R^a^
Cefoxitin, FOX	≤	0.5	S
Chloramphenicol, CHL		64	R
Ciprofloxacin, CIP	≤	0.25	S
Clindamycin, CLN	>	4	R
Erythromycin, ERY	>	8	R
Fusidic acid, FUS	≤	0.5	S
Gentamicin, GEN	≤	1	S
Kanamycin, KAN		64	R
Linezolid, LZD	≤	0.1	S
Mupirocin, MUP	≤	0.5	S
Penicillin, PEN	≤	0.12	S
Synercid, SYN	≤	0.5	S
Rifampicin, RIF	≤	0.016	S
Streptomycin, STR	>	32	R
Sulfamethoxazole, SMX	≤	64	S
Tetracycline, TET	≤	0.5	S
Tiamulin, TIA	≤	0.5	S
Trimethoprim, TMP	≤	2	S
Vancomycin, VAN	≤	1	S
Oxacillin^*^, OXA			S

^a^*S* sensitive, *R* resistant

*antimicrobial sensitivity testing for oxacillin was performed by the disk diffusion method (not a microdilution method)

### Whole genome sequencing (WGS)

Five *S. pseudintermedius* isolates were selected for WGS: four from the present study (VF40237–16-SP1 skin isolate from dam, VF40237–16-SP2 milk isolate from dam, VF40237–16-SP3 isolate from the organs of affected puppy and 7–2018-SP4 isolate subsequently isolated from the dam’s milk before delivering the second litter) and one clinical MRSP as an epidemiologically unrelated isolate (VF27322–15 isolated from a dog with implant). After the extraction of genomic DNA with DNeasy Blood & Tissue Kit (Qiagen, Germany), DNA libraries were prepared using the TruSeq Nano DNA Library Prep Kit (Illumina, USA) for multiplexed paired-end (2 × 150 bp) sequencing. Sequencing was performed on a NextSeq500 platform (Illumina, USA) to an average depth of coverage of 170×. Reads were deposited in the NCBI SRA database under the following BioSample accession numbers: SAMN09781792 (isolate VF40237–16-SP1), SAMN09781793 (isolate VF40237–16-SP2), SAMN09781794 (isolate VF40237–16-SP3), SAMN09781795 (isolate 7–2018-SP4) and SAMN09781796 (isolate VF27322–15).

#### Multilocus sequence typing (MLST)

After performing WGS, the obtained reads were quality-trimmed using the Cutadapt v1.16 (https://cutadapt.readthedocs.io/en/stable/) and assembled using SPAdes v3.11.0 [14]. The assemblies were used as an input for MLST typing according to the *S. pseudintermedius* MLST scheme [15], implemented in the PubMLST *S. pseudintermedius* database (https://pubmlst.org/spseudintermedius/).

#### Identification of acquired resistance genes

The assembled draft genomes were used as an input for the identification of acquired resistance genes using the ResFinder v3.0 (https://cge.cbs.dtu.dk/services/ResFinder/).

#### Single nucleotide polymorphism (SNP)-based phylogeny

The high-quality SNPs were identified in the obtained genomes using the CSI Phylogeny v1.4 pipeline (https://cge.cbs.du.dk/services/CSIPhylogeny/). The maximum-likelihood phylogeny was inferred from the concatenated SNP alignment using the RAxML v8.1.22 [16] under the GTRGAMMA model of nucleotide substitution and 1000 bootstrap replicates.

**Fig. 4:**
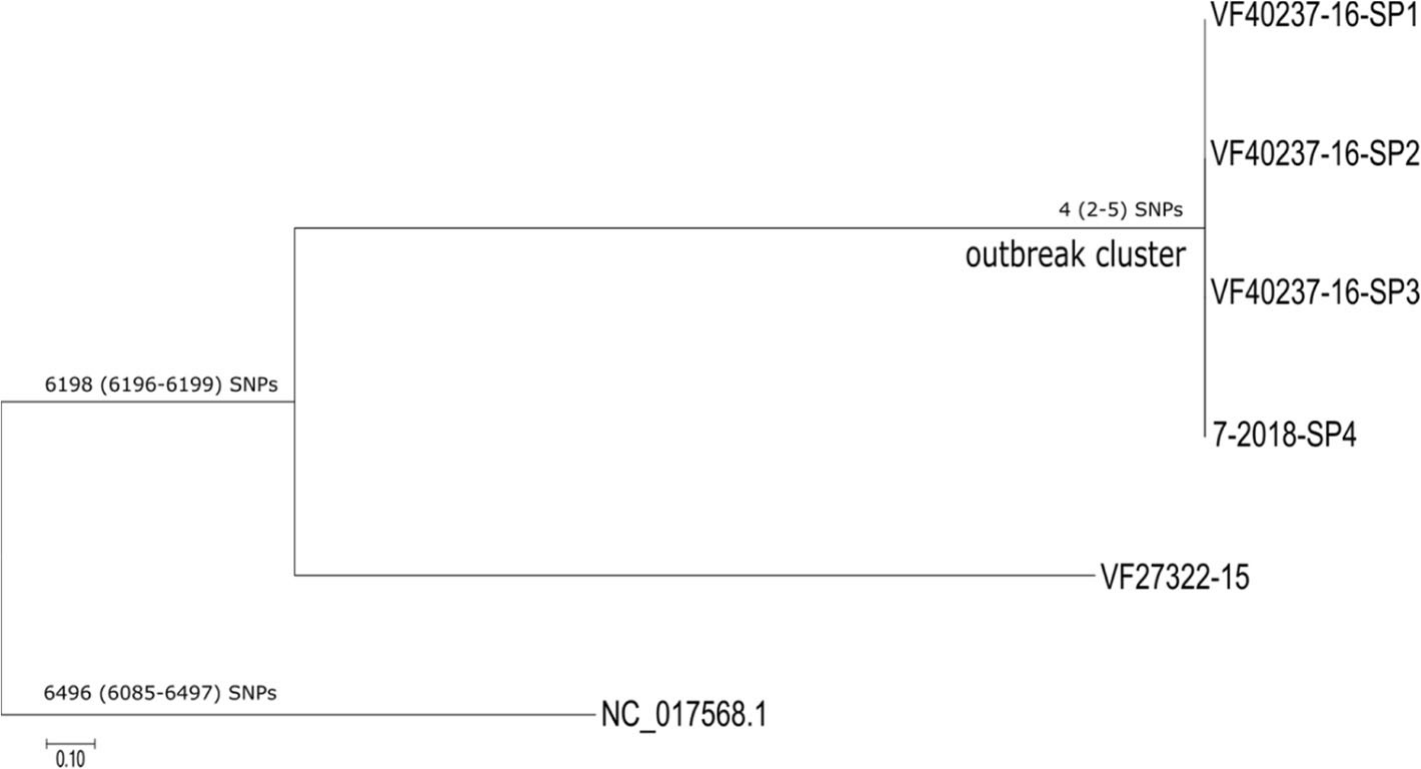
Phylogenetic tree of the examined isolates, constructed from SNPs identified by the CSI Phylogeny pipeline, using NC_007795.1 as the reference. The alignment of the high-quality SNPs contained 9391 sites and the percentage of genomes covered by all isolates was 91.8%. The maximum-likelihood phylogeny was inferred using RAxML v8.1.22 with GTRGAMMA model and 1000 bootstrap replicates. VF40237–16-SP1, VF40237–16-SP2, VF40237–16-SP3, 7–2018-SP4: outbreak-associated clinical isolates; VF27322–15: isolate from a sporadic case of methicillin-resistant *S. pseudintermedius* infection; NC_017568.1: reference isolate. The median, minimum, and maximum pairwise SNP differences among isolates are shown near the root of each cluster, with the minimum and maximum in parentheses. Scale bar, number of substitutions per site

According to the WGS results, the outbreak-related isolates belonged to the MLST sequence type (ST) 241. The following acquired resistance genes were identified in all outbreak-related isolates: (*i*) *cat*(pC221)-like gene (involved in resistance to phenicol), (*ii*) *blaZ* (involved in resistance to beta-lactams), (*iii*) *erm*(B) (involved in resistance to macrolides) and (*iv*) ANT(6)-Ia and Aph(3′)-III (both involved in resistance to aminoglycosides). All the identified resistance genes showed 100% identity and coverage, with the exception of *cat*(pC221)-like gene, which showed 97.7% identity and 100% coverage. The SNP-based analysis further confirmed the epidemiological link between the outbreak-associated strains as they differed in 2–5 SNPs and formed a separate cluster (named ‘outbreak cluster’) on the SNP-based phylogenetic tree (Fig. 4).

## Discussion and conclusions

Neonatal diseases are difficult to diagnose since the clinical signs and results of diagnostic tests are generally non-specific. However, infections are thought to be among the most common causes of perinatal death in dogs [17]. Since most of the dogs are now very well vaccinated, bacterial infections are a more common cause of neonatal death than viral [18]. Puppies are most susceptible to sepsis during the first three weeks of life, with most puppies dying during the first week [19]. Of the Gram-positive bacteria, β-streptococci (usually group G or B) have the potential to cause systemic infections during the first week of life [6, 20]. Despite previous reports, describing puppies dying due to the *S. pseudintermedius* infection [8–10] and *S. pseudintermedius* as the only bacterium isolated from the organs of septicemic puppies and dam’s milk [9], confirmation of the relatedness of the obtained isolates and the transmission of this pathogen through canine milk is still lacking. Even more, despite frequently isolating *S. pseudintermedius* from the canine milk, it was assumed that bacteria from the milk were not the primary cause of septicemia in puppies [11]. In human medicine, there is at least one report of three neonates in which bacteria isolated from the contaminated breast milk caused the neonatal sepsis, even though mothers did not show any clinical signs of a systemic disease or mastitis [21]. In the present paper, similar findings with a fatal septicemia in puppies are reported since the dam did not show any signs of mastitis or other disease.

For clinical cases, collection of samples and their analysis in the microbiological laboratory is important, since the identification of microorganism(s) causing the disease and their susceptibility data guide the selection of the antimicrobial treatment in the patient. However, the disease outcome in the neonates can deteriorate very quickly. Therefore, the empirical antimicrobial treatment with amoxicillin with clavulanic acid is recommended. This class of antibiotics is the safest and still efficacious for many neonatal infections. If one of the puppies from the litter dies, it is of great importance to perform its necropsy since a definitive diagnosis can be obtained and vital information provided for the treatment of the remaining puppies [6].

In the present case of a neonatal sepsis in dogs, the heavy and widespread growth of *S. pseudintermedius* in the internal organs and from the effusions of the dead puppy supported a causative role of *S. pseudintermedius* in the sepsis. WGS of *S. pseudintermedius* isolates obtained from the dam’s milk, skin and organs of the dead puppy showed that the isolates belonged to ST241 and differed in 2–5 SNPs, which confirmed the epidemiological link between the canine milk and the neonatal sepsis in puppies. This sequence type has already been reported in connection to the *S. pseudintermedius* infection, namely for a human clinical case involving an immunocompromised pet owner with two dogs in the same household; both dogs showed no clinical signs of a disease but their nasal samples tested positive for *S. pseudintermedius* ST241 [22]. Thus, *S. pseudintermedius* ST241 found in the present study can be regarded as a *S. pseudintermedius* clone with a proven zoonotic potential.

Our findings point to the conclusion that the dam’s milk is a possible source of pathogenic bacteria that can be transmitted to puppies through weaning. In addition, it can be concluded that *S. pseudintermedius* sepsis can be fatal for the neonates. However, it must be regarded that all of the affected puppies were involved in the same disease episode, but four of them recovered without fatal outcome. Thus, *S. pseudintermedius* cannot be de facto considered as a consistent pathogen of the neonatal sepsis in dogs. Factors that predispose puppies to the bacterial infections and sepsis include the incomplete development, stress, low birth weight, premature neonates and the environmental problems [23], but none of these were observed in the present case.

As in humans, a microbiological screening of the milk from dams is not uniformly recommended, but transmission of microbial agents through the milk of healthy dams should be considered in the neonatal sepsis. An eradicative antibiotic treatment of the *S. pseudintermedius* positive canine mothers and/or temporary discontinuation of breast feeding may represent a relatively simple solution to prevent the recurrent *S. pseudintermedius* sepsis in puppies. Further research is required to characterize the extent and significance of bacterial contamination of the canine breast milk.

## Abbreviations

C-section: Cesarean section
HE: Hematoxylin and eosin
MIC: Minimal inhibitory concentration
MLST: Multilocus sequence typing
MRS: Methicillin-resistant staphylococci
MRSA: Methicillin-resistant *Staphylococcus aureus*
MRSP: Methicillin-resistant *Staphylococcus pseudintermedius*
SNP: Single nucleotide polymorphism
WGS: Whole genome sequencing
